# Predicting protein folding pathways at the mesoscopic level based on native interactions between secondary structure elements

**DOI:** 10.1186/1471-2105-9-320

**Published:** 2008-07-23

**Authors:** Qingwu Yang, Sing-Hoi Sze

**Affiliations:** 1 Department of Computer Science, Texas A&M University, College Station, TX 77843, USA; 2 Department of Biochemistry & Biophysics, Texas A&M University, College Station, TX 77843, USA

## Abstract

**Background:**

Since experimental determination of protein folding pathways remains difficult, computational techniques are often used to simulate protein folding. Most current techniques to predict protein folding pathways are computationally intensive and are suitable only for small proteins.

**Results:**

By assuming that the native structure of a protein is known and representing each intermediate conformation as a collection of fully folded structures in which each of them contains a set of interacting secondary structure elements, we show that it is possible to significantly reduce the conformation space while still being able to predict the most energetically favorable folding pathway of large proteins with hundreds of residues at the mesoscopic level, including the pig muscle phosphoglycerate kinase with 416 residues. The model is detailed enough to distinguish between different folding pathways of structurally very similar proteins, including the streptococcal protein G and the peptostreptococcal protein L. The model is also able to recognize the differences between the folding pathways of protein G and its two structurally similar variants NuG1 and NuG2, which are even harder to distinguish. We show that this strategy can produce accurate predictions on many other proteins with experimentally determined intermediate folding states.

**Conclusion:**

Our technique is efficient enough to predict folding pathways for both large and small proteins at the mesoscopic level. Such a strategy is often the only feasible choice for large proteins. A software program implementing this strategy (SSFold) is available at .

## Background

As early studies revealed that an unfolded protein can fold spontaneously to a three-dimensional structure under suitable environmental conditions [1,2], traditional approaches to understanding protein folding have focused on the prediction of the native structure. As more studies showed the existence of intermediates and interaction among residues during the protein folding process [3,4], there is substantial interest to understand the time order of events during the formation of the tertiary structure. From the free energy point of view, each conformation of a protein is associated with a free energy and the protein folds from the high-energy denatured conformation to its folded structure along a funnel-like energy landscape [5,6].

Although advances in experimental techniques allow the investigation of protein folding pathways at the microsecond timescale [7,8], experimental determination of protein folding pathways remains difficult. Most studies are only able to identify general characteristics of the folding pathway without much details and are limited to analyzing small proteins. Computational techniques are often used to simulate protein folding and the problem is transformed to energetic optimization problems, that is, computational search for global energy minimum over all possible conformations. The most accurate computational techniques utilize molecular dynamics to determine the order of events that lead to the tertiary structure through atomic-level simulations [9-12]. Due to the extremely large conformation space, these approaches suffer from well-known problems accompanying high dimensionality, including computational expensiveness and ease of trapping in local minima, and are applicable only to small proteins in a short time course.

**Figure 1:**
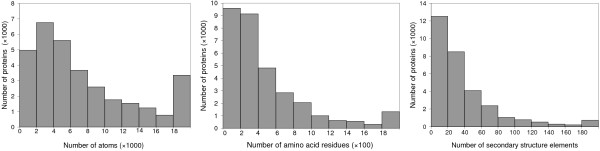
**The distribution of the number of atoms, the number of amino acid residues, and the number of secondary structure elements among 32237 protein structures in the Protein Data Bank (PDB) **[21]. Each bar (except the rightmost one in each chart) shows the number of proteins that have values falling between the indicated lower and upper limits. The rightmost bar in each chart shows the number of proteins that have values of at least the indicated lower limit.

By omitting some details, proteins can be represented at the level of amino acids. Kolinski and Skolnick [13] performed Monte Carlo simulations of protein folding on a reduced lattice representation of the protein *α*-carbon backbone. Yue and Dill [14] limited the conformation space to a discrete subset of possibilities and used a branch-and-bound procedure to search for near-optimal conformations. Alm and Baker [15] and Muñoz and Eaton [16] further observed that the availability of the known native structure can dramatically reduce the search space. Alm and Baker [15] took into account only native interactions among residues and used a sequential binary collision model to predict protein folding mechanisms from the perspectives of free energy landscapes, while Muñoz and Eaton [16] used a slightly different approach of employing distinct free energy costs for different secondary structures. Amato and Song [17] represented a protein by the torsional angles of its residues and used the probabilistic roadmap technique with a biased sampling strategy around the native structure to predict folding pathways and secondary structure formation order. Liwo et al [18] and Kmiecik and Kolinski [19,20] showed that the use of reduced models of proteins is highly successful in characterizing folding pathways for small proteins at the mesoscopic level. Although these techniques are able to predict folding pathways very accurately for proteins with up to about 100 residues, the majority of proteins in the Protein Data Bank (PDB) [21] are much larger (Figure 1).

The problem with representing a protein at the amino acid level is that even with the assumption that each residue has only two states (ordered or disordered), a protein with *n *residues still has 2^*n *^possible conformations [15]. To overcome this problem, several recent approaches represent a protein at the level of secondary structure elements (SSEs), in which each element corresponds to one helix or one *β*-strand. By adopting the framework model in which secondary structures are thought to fold relatively independently of the tertiary structure [22], each SSE is treated as an indivisible unit that interacts with other SSEs as a whole. Since the number of SSEs in a protein is small (Figure 1), this model is much more tractable to simulate. Eyrich et al [23] assumed that the SSEs are fixed and used a branch-and-bound algorithm to search for near-optimal tertiary structures. Apaydin et al [24] assumed that each SSE of a protein is already in native conformation and moves as a unit, and used the probabilistic roadmap approach to predict folding pathways. Zaki et al [25] proposed an algorithm to predict unfolding pathways based on applying a minimum cut procedure to a weighted graph that represents a protein's contact map or interaction strength between SSEs. Although the underlying assumption that intermediate secondary structures are fully folded before the formation of tertiary structures is not satisfied for most proteins, these studies show that such a strategy is sufficient to study protein folding pathways at the mesoscopic level.

In this paper, our goal is to further reduce the conformation space without sacrificing prediction accuracy. This is achieved by assuming that SSEs that do not yet interact with each other are independent and can be treated separately. A conformation is represented by a collection of fully folded structures in which each of them contains a set of interacting SSEs. By using a steepest descent strategy, we show that it is possible to predict the most energetically favorable folding pathway of large proteins with hundreds of residues at the mesoscopic level and this model is detailed enough to distinguish between different folding pathways of structurally very similar proteins. In difference from the technique in [24], we do not consider the spatially moving process before the SSEs form native contacts, and thus we are able to achieve much better computational efficiency.

## Methods

Assume that the native structure of a protein is known. The protein folding pathway prediction problem is to find an ordered sequence of intermediate conformations to fill the gap between the unfolded state and the native tertiary structure. At the secondary structure level, a protein can be viewed as an ordered sequence of secondary structure elements (SSEs) interspersed with irregular turns or loops, where each SSE is either a helix or a *β*-strand, and each *β*-sheet consists of a variable number of *β*-strands that are not necessarily consecutive on the primary sequence. We represent each protein by *t*_0_*s*_1_*t*_1_⋯*s*_*k*_*t*_*k*_, where *k *is the number of SSEs, *s*_*i *_denotes the *i*th SSE, *t*_*j *_denotes the *j*th turn, and these elements are in the same order as they appear on the primary sequence. Given the three-dimensional structure of a protein, the assignment of SSEs can be obtained directly from the Protein Data Bank (PDB) [21] or using programs such as DSSP [26].

Following [24] and [25], we consider each SSE as an indivisible unit that folds independently of the others according to the contacts present in the native structure. This is based on the framework model that assumes that extensive intermediate secondary structures exist before they are assembled into the tertiary structure [22], and our goal is to predict the interaction order of SSEs during folding. Based on the observation in [15] and [16] that a model using only native interactions can explain most experimental results, we assume that the interactions between SSEs or turns are the same as the ones present in the native structure. Although these assumptions are often not satisfied as there are many proteins in which there are no clear secondary structures before the formation of tertiary structures or there are no clear preservations of secondary structures throughout folding, such a strategy is sufficient for studying folding pathways at the mesoscopic level and is often the only feasible choice for large proteins.

**Figure 2:**
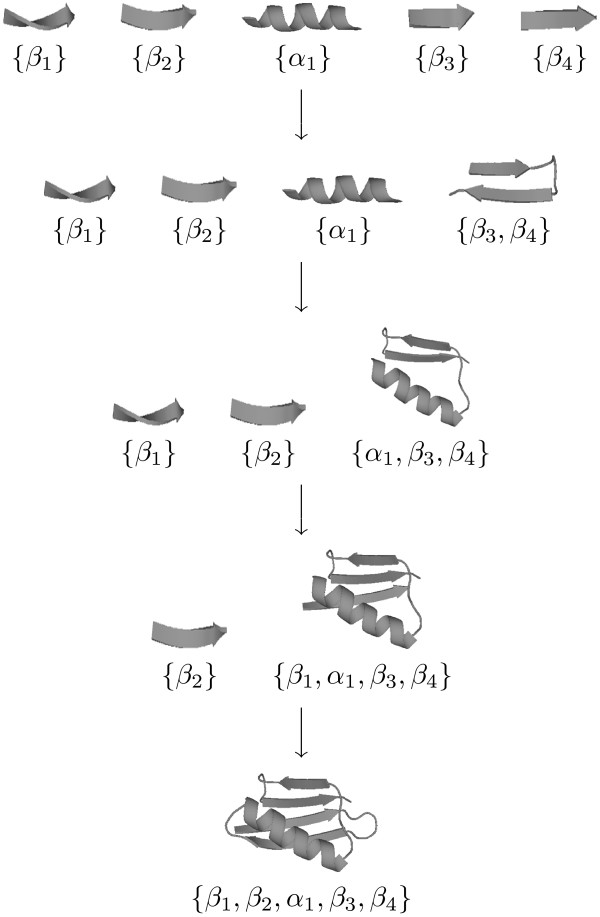
**Illustration of the folding pathway prediction for GB1**. The starting conformation {{*β*_1_}, {*β*_2_}, {*α*_1_}, {*β*_3_}, {*β*_4_}} corresponds to the initial state. There are three intermediate conformations in the predicted folding pathway, including {{*β*_1_}, {*β*_2_}, {*α*_1_}, {*β*_3_, *β*_4_}}, {{*β*_1_}, {*β*_2_}, {*α*_1_, *β*_3_, *β*_4_}}, and {{*β*_2_}, {*β*_1_, *α*_1_, *β*_3_, *β*_4_}}. The ending conformation {{*β*_1_, *β*_2_, *α*_1_, *β*_3_, *β*_4_}} corresponds to the native state.

We represent a conformation of a protein on the folding pathway by *C *= {*S*_1_, ..., *S*_*k*_}, where each *S*_*i *_represents a structure consisting of a set of fully folded SSEs and there are no interactions between two different sets *S*_*j *_and *S*_*j*' _(see Figure 2 for an illustration). Since our focus is on the SSEs, turns are not included in the conformation but will be utilized when computing energies (see below). The protein folding problem is transformed to identifying a sequence of conformational changes that start from an initial state with fully folded SSEs but no interactions between SSEs through some intermediate conformations and ending in the native structure (Figure 2). Each conformational change corresponds to finding a new pair of interactions that merges two smaller structures of SSEs into a bigger one. Figure 2 illustrates the folding pathway prediction on the B1 domain of the streptococcal protein G (GB1). In the prediction, *β*_3 _and *β*_4 _interact first, then *α*_1 _is added, followed by *β*_1 _and *β*_2_.

Folding pathway predictions are obtained through the computation of free energies of intermediate conformations. For an intermediate conformation *C *= {*S*_1_, ..., *S*_*k*_}, the free energy *E*(*C*) of *C *is defined as:

$$E(C)=\sum_{i=1}^{k}E(S_{i}),$$

where each *S*_*i *_is viewed as an isolated entity and each *E*(*S*_*i*_) is obtained separately by extracting the three-dimensional coordinates of its residues from the Protein Data Bank (PDB) [21] and using the Rosetta software [27] to compute its free energy. The original Rosetta energy function is used, which is obtained by representing each side chain by a centroid that is located at the center of mass, and computing a weighted sum of the binned probability descriptions of multiple effects, including the solvation and electrostatic effects based on observed distributions in known protein structures, the secondary structure packing effects that include strand pairing, strand arrangement into sheets and helix-strand packing, and the effects of steric repulsion and Van der Waals interactions (more details are available in [28] and in Table I of [27]). To take the backbone into consideration, a turn *t*_*j *_is included in the computation of *E*(*S*_*i*_) if both of its adjacent SSEs *s*_*j *_(if it exists) and *s*_*j*+1 _(if it exists) are included in *S*_*i*_.

**Figure 3:**
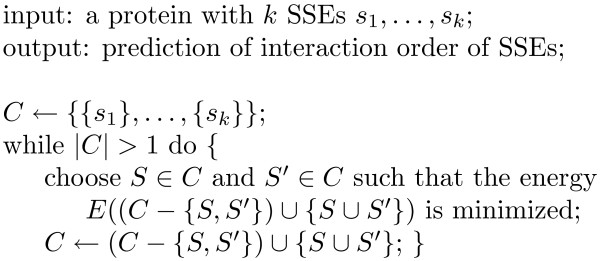
**Algorithm SSFold to predict the most energetically favorable interaction order of SSEs that corresponds to a folding pathway**. Each iteration corresponds to a conformational change that results from a new pair of interactions. Within a folded structure, a turn is included in the energy computations only when adjacent SSEs are included in the structure.

Since the interactions that favor folding usually decrease the free energy while the interactions that destabilize the native structure increase the free energy, our goal is to find the most energetically favorable folding pathway by identifying the conformational change that decreases the free energy the most in each step so that the protein can get to lower energy states as quickly as possible. Figure 3 illustrates our SSFold algorithm that uses a steepest descent strategy to choose a new pair of interactions that leads to a conformation with the lowest free energy in each iteration. This procedure is very efficient since only *k *- 1 iterations are needed. Within each iteration, *O*(*k*^2^) comparisons are needed to find the best pair of interactions that results in the lowest free energy. This leads to an overall time complexity of *O*(*k*^3^*t*), where *k *is the number of SSEs in a protein and *t *is the time to compute the free energy of a potentially partial protein that contains only some of the SSEs and turns.

## Results

We test our strategy on proteins from the Protein Data Bank (PDB) [21] that have known intermediate folding states from experimental data. We illustrate that our model is detailed enough to distinguish between subtle differences in the folding pathways of the streptococcal protein G, the peptostreptococcal protein L, and variants NuG1 and NuG2 of protein G, which are all structurally very similar proteins. We demonstrate that our approach is applicable to large proteins with hundreds of residues by testing it on the 416 residue pig muscle phosphoglycerate kinase (PGK). We further test it on proteins studied in [29] and [25] to validate that our model has very good accuracy.

### Proteins GB1, LB1, NuG1 and NuG2

The 56 residue B1 immunoglobulin binding domain of streptococcal protein G (GB1, PDB: 1GB1) and the 62 residue B1 immunoglobulin binding domain of peptostreptococcal protein L (LB1, PDB: 2PTL) have been used extensively as model systems for studying protein folding mechanisms [30-37]. Both GB1 (see Figure 2) and LB1 consist of one *β*-sheet with four strands and one *α*-helix. Strands 1 and 2 form an N-terminal *β*-hairpin, while strands 3 and 4 form a C-terminal *β*-hairpin. Although GB1 and LB1 have very similar tertiary structures, they have different folding pathways. As suggested by [29], a detailed model is needed to distinguish between them.

**Figure 4:**
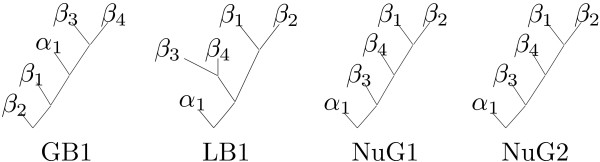
**Folding pathway predictions for GB1, LB1, NuG1 and NuG2**. Each internal node represents a new pair of interactions and nodes that are higher in the tree indicate earlier interactions. Also compare to Figure 2 for GB1.

Figure 4 shows our folding pathway predictions for GB1 and LB1 (see also Figure 2 for GB1).

Experimental results showed that the C-terminal *β*-hairpin in GB1 is formed in the transition state of the folding pathway and serves as the starting point on which the rest of the protein can fold [35]. Similar results were obtained using the diffusion-collision model [38]. Our prediction is consistent with these results. In contrast, experimental results showed that only the N-terminal *β*-hairpin in LB1 is mainly formed in the transition state and non-random structures can be detected in the region [34,39]. Our algorithm also predicts that the N-terminal *β*-hairpin forms earlier than the C-terminal *β*-hairpin in LB1.

Two protein G variants, NuG1 (PDB: 1MHX) and NuG2 (PDB: 1MI0), were designed to have a different folding mechanism from protein G by replacing some residues of protein G [36]. In NuG1 and NuG2, the stability of the N-terminal *β*-hairpin is enhanced while the stability of the C-terminal *β*-hairpin is reduced, with the N-terminal *β*-hairpin forming contacts earlier than the C-terminal *β*-hairpin in both cases [36].

Thomas et al [40] showed that it is more difficult to distinguish between the folding pathways of protein G and its variants NuG1 and NuG2 than to distinguish between the folding pathways of protein G and protein L. In our predictions in Figure 4, NuG1 and NuG2 have the same folding pathway, with the N-terminal *β*-hairpin folded first. This is consistent with the experimental results in [41] and the predictions in [40].

**Figure 5:**
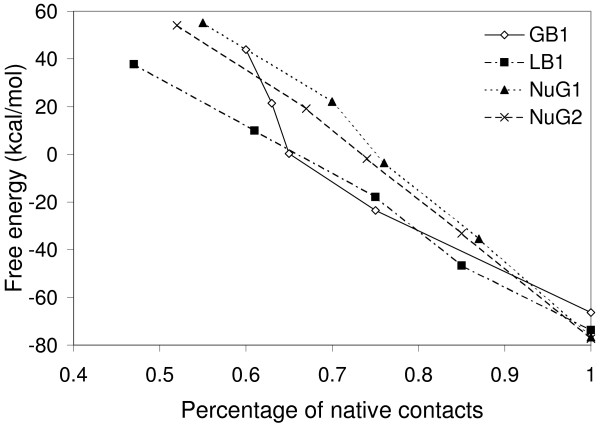
**Free energy profiles of GB1, LB1, NuG1 and NuG2 in our predictions**. A native contact is defined to be a pair of amino acids that have their *α*-carbon atoms within 7 Å of each other. Each starting point corresponds to the initial state in which each SSE has already completed its native fold independently and there are no interactions between SSEs.

Figure 5 shows the free energy profiles of GB1, LB1, NuG1 and NuG2 in our predictions. Our predicted folding pathway of GB1 is a non-frustrated curve, similar to the average macroscopic folding pathway given by [37]. When compared to GB1, NuG1 and NuG2 have similar profiles and higher initial free energy, but their native structures have lower free energy and are more stable, which is consistent with the analysis in [41].

### Pig muscle PGK: a large protein

Phosphoglycerate kinase (PGK) from various organisms has been used as a model system for studying domain-domain interactions of multiple-domain proteins [42-44]. The pig muscle PGK (PDB: 1KF0) [43] is a large two-domain protein with 416 residues, with the N-terminal domain consisting of residues 1 to 155 and the C-terminal domain consisting of residues 156 to 416. There are 21 *α*-helices and 17 *β*-strands, which belong to four different *β*-sheets A, B, C and D, arranged as follows on the primary sequence: *α*_1 _*β*_A4 _*α*_2 _*α*_3 _*β*_A3 _*α*_4 _*β*_A1 _*α*_5 _*β*_A2 _*α*_6 _*β*_B1 _*β*_B2 _*α*_7 _*β*_A5 _*α*_8 _*α*_9 _*β*_A6 _*α*_10 _*β*_C3 _*α*_11 _*α*_12 _*β*_C2 _*α*_13 _*α*_14 _*α*_15 _*β*_C1 _*β*_D2 _*β*_D1 _*β*_D3 _*α*_16 _*β*_C4 _*α*_17 _*α*_18 _*β*_C5 _*α*_19 _*β*_C6 _*α*_20 _*α*_21_.

**Figure 6:**
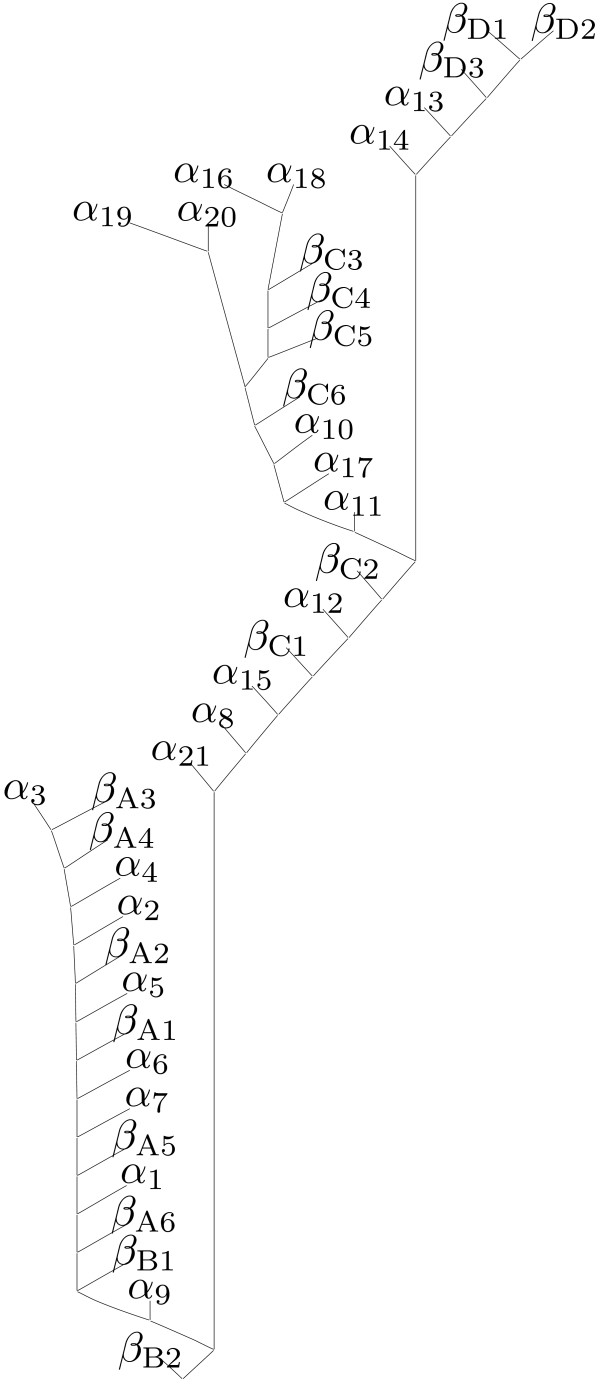
Folding pathway prediction for the pig muscle PGK.

Figure 6 shows our folding pathway prediction for the pig muscle PGK, in which *β*-sheet D is formed first, followed by the formation of *β*-sheet C interspersed with *α*-helices in the C-terminal domain. After most SSEs of the C-terminal domain are formed, the SSEs of the N-terminal domain begin to form, with *β*-sheet A formed before *β*-sheet B interspersed with *α*-helices in the N-terminal domain.

Szilágyi and Vas [45] suggested a sequential domain refolding mechanism for the pig muscle PGK, in which folding of the C-terminal domain is independent of the N-terminal domain and takes place first, and folding of the N-terminal domain starts after most of the C-terminal domain folds. The authors also suggested that an intermediate consists of a folded C-terminal domain and a still unfolded N-terminal domain. Our prediction is consistent with these experimental results.

### Other proteins

**Figure 7:**
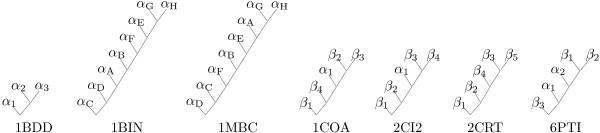
Folding pathway predictions for *Staphylococcus aureus* protein A domain B (PDB: 1BDD), leghemoglobin A (PDB: 1BIN), myoglobin (PDB: 1MBC), chymotrypsin inhibitor 2 structure 1 (PDB: 1COA), chymotrypsin inhibitor 2 structure 2 (PDB: 2CI2), cardiotoxin III (PDB: 2CRT), and bovine pancreatic trypsin inhibitor BPTI (PDB: 6PTI).

Figure 7 shows folding pathway predictions for various small proteins that have known intermediate folding states from biological experiments. The proteins 1BDD and 2CRT were studied in [29], while the proteins 1BIN, 1MBC, 2CI2 and 6PTI were studied in [25].

The B domain of *Staphylococcus aureus *protein A (PDB: 1BDD) consists of three *α*-helices. In our prediction, *α*_2 _and *α*_3 _interact first, then *α*_1 _is added. This is consistent with the result of the out-exchange experiment in [46] and experimental results under high temperature [47].

Although two members of the globin protein family, leghemoglobin A (PDB: 1BIN) and myoglobin (PDB: 1MBC), have very low sequence similarity, they both consist of eight *α*-helices and have very similar tertiary structures. Nishimura et al [48] compared their folding pathways experimentally. For leghemoglobin A, *α*_G_, *α*_H_, and part of *α*_E _form stable structures first, while *α*_A _and *α*_B _form in the later stages of the folding pathway. For myoglobin, *α*_A_, *α*_G _and *α*_H _form stable contacts first. The main difference between the two folding pathways is that *α*_A _and *α*_B _form earlier in the folding pathway of myoglobin than in the folding pathway of leghemoglobin A [48]. Our predictions are able to distinguish between these subtle differences. For leghemoglobin A, *α*_G _and *α*_H _are predicted to interact first, then *α*_E _is added, with *α*_B _and *α*_A _added later. For myoglobin, *α*_G _and *α*_H _are also predicted to interact first, then *α*_A _is added, followed by *α*_E _and *α*_B_.

There are two crystal structures for chymotrypsin inhibitor 2 (PDB: 1COA and 2CI2). While 2CI2 consists of 83 residues, 1COA is a fragment of 2CI2 from residues 20 to 83. They both consist of one *α*-helix and four *β*-strands, which are arranged as *β*_1_*α*_1_*β*_2_*β*_3_*β*_4 _in 1COA and *β*_1_*α*_1_*β*_4_*β*_3_*β*_2 _in 2CI2. In our predictions, 1COA and 2CI2 have the same folding pathway, with the middle two *β*-strands interacting first, then the *α*-helix is added, followed by the C-terminal *β*-strand, and the N-terminal *β*-strand is added last. For 1COA, simulation by [49] demonstrated that *β*_2 _and *β*_3 _form contacts first, then *α*_1 _is added to form a folding nucleus. The coalescence of *β*_1 _is the rate-limiting step and is completed at the end of the folding process. This is consistent with the result of the out-exchange experiment in [46] that showed that *β*_2_, *β*_3 _and *α*_1 _form contacts first. Our prediction is consistent with these results.

The all *β*-sheet protein cardiotoxin III (PDB: 2CRT) consists of five strands. While *β*_1 _and *β*_2 _form a double-stranded domain, *β*_3_, *β*_4 _and *β*_5 _form a triple-stranded domain. By the amide proton pulse exchange experiment, Sivaraman et al [50] showed that the triple-stranded domain forms earlier than the double-stranded domain during the refolding process. The carbonyl groups in *β*_3 _and the amide groups in *β*_5 _form hydrogen bonding partners, which are important for the formation of a hydrophobic cluster [50]. Our prediction is consistent with these results, with *β*_3 _and *β*_5 _interacting first, then *β*_4 _is added to form the triple-stranded domain, followed by *β*_2 _and *β*_1 _in the double-stranded domain.

Bovine pancreatic trypsin inhibitor BPTI (PDB: 6PTI) is a globular protein with two *α*-helices and three *β*-strands, which are arranged as *α*_1_*β*_2_*β*_1_*β*_3_*α*_2_. Three disulfide bonds between residues 5 and 55, 14 and 38, and 30 and 51 play an important role in stabilizing the native structure [51], and their formation order was studied in [52]. In our prediction, *β*_1 _and *β*_2 _interact first, then *α*_2 _is added. This brings residues 30 and 51 close together and helps to form the disulfide bond between them. Then *α*_1 _is added and this helps to form the disulfide bond between residues 5 and 55, and 14 and 38. Our prediction that *β*_1 _and *β*_2 _interact earlier than the two *α*-helices is consistent with the result in [53].

## Discussion

While our strategy corresponds most closely to the diffusion-collision model that allows folding to proceed independently in different parts of a protein [54], it is possible to use a modified strategy for other models. For example, to simulate the nucleation-propagation model [55] or the nucleation-condensation model [56], in which the existence of a nucleus facilitates further folding, one can iteratively add a SSE that results in the lowest free energy to the nucleus. Since energy computations can still be slow and can take hours, which account for significant amount of computation time in our algorithm, it is also possible to use lower resolution methods to compute energy.

While our strategy finds the most energetically favorable protein folding pathway, there are evidences that multiple folding pathways exist [5,57]. The ability to analyze multiple folding pathways will also allow the study of protein misfolding [58]. Our approach can be generalized to study the entire free energy landscape [5] as follows: construct a graph in which each vertex represents a biologically plausible conformation and each edge represents a feasible conformation change, which is similar to the roadmap graph in [24] and [17] and the protein folding network in [59] except that we consider each SSE as an indivisible unit. Various graph-theoretic algorithms can then be used to generate predictions of alternative folding pathways.

## Conclusion

We have shown that our procedure has sufficient accuracy to distinguish between subtle differences and our strategy can be applied to large proteins due to its speed. An important future direction is to consider cooperative folding of secondary structures without too much sacrifice in speed, that is, when folding in one secondary structure affects folding in others.

## Authors' contributions

QY performed the research and implemented the algorithm. S–HS supervised the research. All authors read and approved the final manuscript.
